# Reversible electric-field-induced phase transition in Ca-modified NaNbO_3_ perovskites for energy storage applications

**DOI:** 10.1038/s41598-023-33975-6

**Published:** 2023-04-25

**Authors:** Seiyu Aso, Hiroki Matsuo, Yuji Noguchi

**Affiliations:** 1 Department of Computer Science and Electrical Engineering, Graduate School of Science and Technology, Kumamoto University, 2-39-1, Kurokami, Chuo-ku, Kumamoto, 860-8555 Japan; 2 International Research Organization for Advanced Science & Technology (IROAST), Kumamoto University, 2-39-1, Kurokami, Chuo-ku, Kumamoto, 860-8555 Japan; 3 Division of Information and Energy, Faculty of Advanced Science and Technology, Kumamoto University, 2-39-1, Kurokami, Chuo-ku, Kumamoto, 860-8555 Japan

**Keywords:** Energy science and technology, Materials science, Materials for devices

## Abstract

Sodium niobate (NaNbO_3_) is a potential material for lead-free dielectric ceramic capacitors for energy storage applications because of its antipolar ordering. In principle, a reversible phase transition between antiferroelectric (AFE) and ferroelectric (FE) phases can be induced by an application of electric field (*E*) and provides a large recoverable energy density. However, an irreversible phase transition from the AFE to the FE phase usually takes place and an AFE-derived polarization feature, a double polarization (*P*)-*E* hysteresis loop, does not appear. In this study, we investigate the impact of chemically induced hydrostatic pressure (*p*_chem_) on the phase stability and polarization characteristics of NaNbO_3_-based ceramics. We reveal that the cell volume of Ca-modified NaNbO_3_ [(Ca_*x*_Na_1−2*x*_*V*_*x*_)NbO_3_], where *V* is A-site vacancy, decreases with increasing *x* by a positive *p*_chem_. Structural analysis using micro-X-ray diffraction measurements shows that a reversible AFE–FE phase transition leads to a double *P*-*E* hysteresis loop for the sample with *x* = 0.10. DFT calculations support that a positive *p*_chem_ stabilizes the AFE phase even after the electrical poling and provides the reversible phase transition. Our study demonstrates that an application of positive *p*_chem_ is effective in delivering the double *P*-*E* loop in the NaNbO_3_ system for energy storage applications.

## Introduction

Because of the increasing demand for electrical energy storage and conversion applications, rechargeable energy storage components such as batteries, electrochemical capacitors, and dielectric capacitors have been intensively studied^1–3^. Dielectric capacitors have various advantages from the viewpoints of high power density, fast charge/discharge capability, long work lifetime, and high-temperature stability^4,5^. Therefore, they are the optimal option for applications in pulsed-discharge and power-conditioning systems, high-powered accelerators, and self-powered IoT devices^6,7^.

To achieve a high recoverable energy density for dielectric capacitors, the following polarization (*P*)-electric field (*E*) properties are advantageous: a smaller remanent polarization (*P*_r_) and a larger maximum polarization (*P*_max_). Antiferroelectric (AFE) materials are a leading candidate, because their antipolar ordering of constituting atoms enables us to obtain zero *P*_r_ and a markedly large *P*_max_^8,9^. When an *E* is applied and its strength exceeds a threshold (*E*_T_), the antipolar polarization changes to a ferroelectric (FE) one with a robust polarization; given that the field is turned off, the AFE phase with zero *P*_r_ reappears. This reversible *E*-induced AFE-FE phase transition provides the characteristic double *P*-*E* loop^10–12^. However, there are limited reports of the perovskite oxides exhibiting the double *P*-*E* hysteresis loop at ambient conditions. This is because the *E*-induced phase transition is irreversible or cannot be achieved owing to *E*_T_ greater than their breakdown fields.

**Figure 1:**
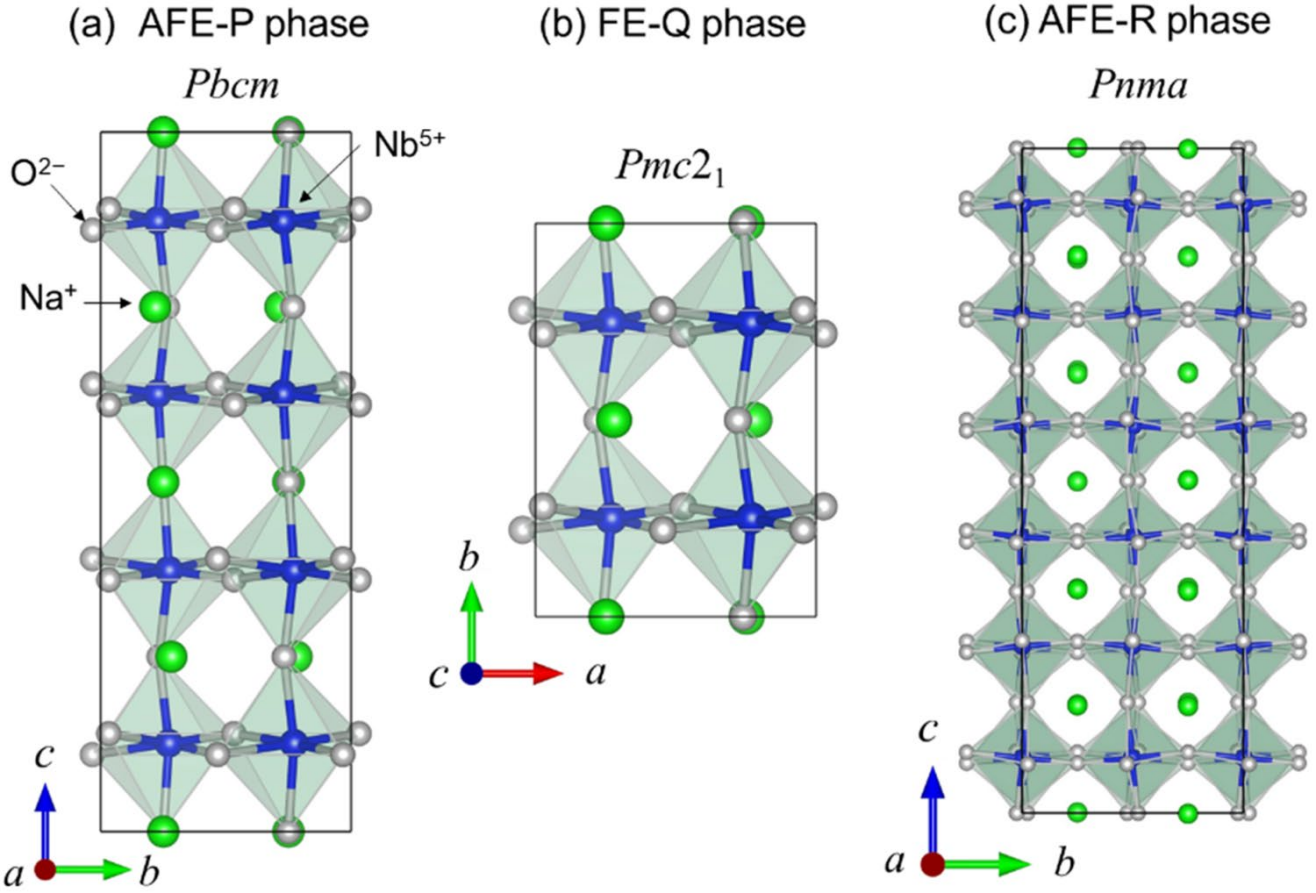
Crystal structures of NaNbO_3_: (**a**) AFE-P (*Pbcm*) phase, (**b**) FE-Q (*Pmc*2_1_) phase and (c) AFE-R (*Pnma*) phase. The green, blue, and gray balls indicate Na^+^, Nb^5+^ and O^2−^, respectively. The data of the AFE-P, FE-Q and AFE-R phases are referenced from Ref.^51^ (ICDD No. 00–033–1270),^52^ (ICDD No. 01–079–7429) and^15^ (COD No. 4329527), respectively.

NaNbO_3_ is one of the potential lead-free AFE perovskite oxides; it crystalizes in orthorhombic *Pbcm* (P phase) with the √2 × √2 × 4 superlattice of the primitive cell of simple perovskite structure in the temperature (*T*) range of −100 °C < *T* < 373 °C and shows a complicated phase transition behavior^13–15^. Though the antipolar ordering was reported in the 1960s and 1970s^16,17^ (Fig. 1a), the double *P*-*E* hysteresis loop was not obtained, because an application of *E* caused an irreversible phase transition and a resultant orthorhombic *Pmc*2_1_ (FE) phase (Q phase with a 2 × √2 × √2 superlattice of the primitive cell) appears^18,19^ (Fig. 1b). Once the FE-Q phase is induced at fields exceeding *E*_T_, the FE-Q phase remains stable even after removing *E*, i.e., the AFE-P phase is not recovered^18,20,21^. This irreversible phase transition from the AFE-P to the FE-Q phase is due to a small free-energy difference between them.

Inspired by materials design in PbZrO_3_-based AFEs^22–26^, the formation of solid solutions and chemical doping have been adopted for NaNbO_3_^20,27–30^. Empirically, the simultaneous substitutions of smaller cations on the Na site and larger cations on the Nb site reduce the Goldschmidt tolerance factor and stabilize the AFE-P phase^31^. Zhang et al. have reported that 0.95NaNbO_3_–0.05SrSnO_3_ with a tolerance factor of 0.964, which is slightly smaller than that of NaNbO_3_ (0.967), exhibits reversible *E*-induced phase transitions as a result of an expansion of unit cell volume accompanied by disordered Na atoms^32^. NaNbO_3_–CaZrO_3_ is also a representative solid solution showing the double *P*-*E* hysteresis loop. Shimizu *et al.* have reported that the substitutions of Ca^2+^ on the Na site and Zr^4+^ on the Nb site lower the polarizability and reduce the tolerance factor while maintaining charge neutrality. This materials modification leads to a reversible *E*-induced phase transition for the 0.96NaNbO_3_–0.04CaZrO_3_ composition^31^.

It is interesting to note that chemically induced hydrostatic pressure (*p*_chem_) is an important degree of freedom for manipulating materials properties^33^. It is defined that *p*_chem_ is the volume-averaged lattice internal force caused by chemical modifications. Various functional materials such as superconductors^34,35^, ferroelectrics^33,36,37^, and negative/zero thermal expansion materials^38,39^ etc. exhibit structures and properties tuned by *p*_chem_. A straightforward approach to apply *p*_chem_ to the NaNbO_3_ lattice (pseudocubic unit cell volume *V*_pc_ = 5.96 × 10^−2^ nm^3^) is to form a solid solution with counterparts with different *V*_pc_. The choice of perovskites with a smaller *V*_pc_ leads to lattice shrinkage, which can be regarded as an effect of positive *p*_chem_, while that with a larger *V*_pc_ results in a lattice expansion caused by negative *p*_chem_.

The formation of solid solutions with the counterparts having a larger *V*_pc_ such as SrSnO_3_^32^ (*V*_pc_ = 6.56 × 10^−2^ nm^3^^40^), CaZrO_3_^31,41^ (6.46 × 10^−2^ nm^3^^42^), CaHfO_3_^43^ (6.39 × 10^−2^ nm^3^^44^) and CaSnO_3_^45^ (6.21 × 10^−2^ nm^3^^46^) can be regarded as an application of negative *p*_chem_ into the NaNaO_3_ lattice. The fine tuning of the compositions results in a reversible *E*-induced phase transition. In contrast, There are few reports on the reversible phase transition for the counterparts with a smaller *V*_pc_^47,48^. These results suggest that a positive *p*_chem_ is not suitable for the chemical modification.

For the above-mentioned systems, the Na site as well as the Nb site involve foreign atoms to satisfy charge neutrality. This type of modifications is likely to vary *p*_chem_ but indeed disturbs the Nb-O-Nb covalent bonding, more generally, an orbital hybridization between niobium 4*d* and oxygen 2*p*, that plays a central role in ferroelectricity^49,50^. To elucidate the influence of *p*_chem_ in a strict manner, the lattice needs to be modified only on the Na site while the Nb site remains intact.

In this paper, we report the impact of *p*_chem_ on the crystal structure and polarization properties of NaNbO_3_ by a substitution only on the Na site. To apply *p*_chem_, the Na site is partially occupied by Ca^2+^ and Na vacancy (*V*) while the Nb site remains unchanged; this chemical tunning is termed ‘Ca modification’ with the following formula (Ca_*x*_Na_1−2*x*_*V*_*x*_)NbO_3_. Our combined investigation by ceramic experiments and density functional theory (DFT) calculations shows that the Ca-modified ceramic sample with a reduced *V*_pc_ exhibits a double *P*-*E* hysteresis loop as a result of the stabilization of the AFE-P phase. We demonstrate that a positive *p*_chem_ derives the AFE-P phase and delivers the reversible *E*-induced phase transition.

## Results

**Figure 2:**
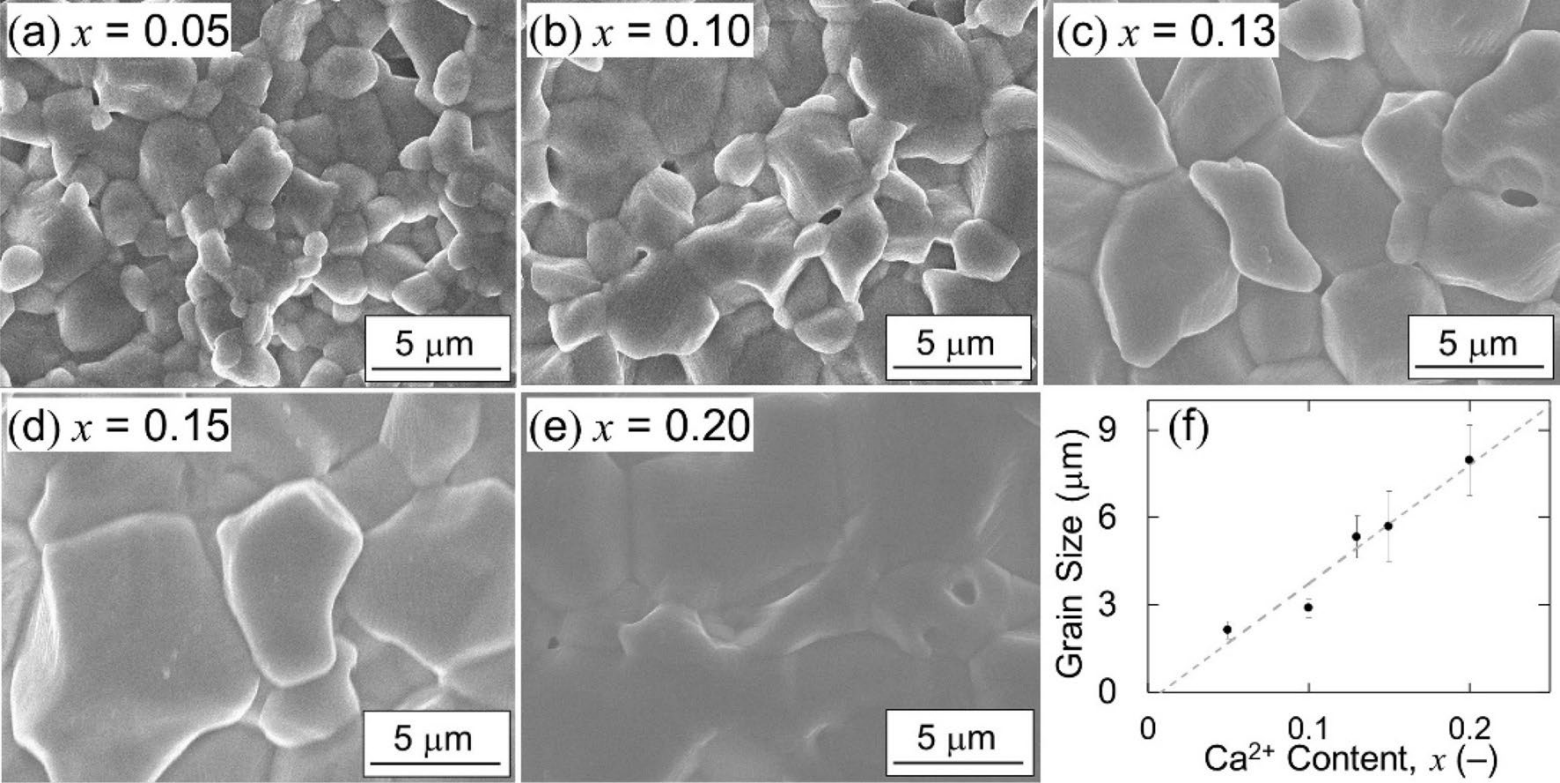
(**a**)–(**e**) Cross sectional SEM images of the Ca-modified samples with *x* of (**a**) 0.05, (**b**) 0.10, (**c**) 0.13, (**d**) 0.15, and (**e**) 0.20. (**f**) Composition dependence of the average grain size estimated by the intercept method using the lower magnification images shown in Fig. S1.

Figure 2 shows the scanning electron microscope (SEM) images of the Ca-modified NaNbO_3_ ceramics. All the samples have dense microstructures; the average grain size is 2.1 µm for *x* = 0.05 and increases with increasing *x* (7.9 µm for *x* = 0.20) (Fig. 2f). The increasing tendency of the grain size and the relative density with increasing *x* indicate that the Ca modification promotes grain growth. We note that the average grain sizes are much larger than a critical grain size of 0.27 μm below which the FE-Q phase is stabilized by intragranular stress^53^. We think that the grain size effect on the phase stability can be neglected in our samples.

**Figure 3:**
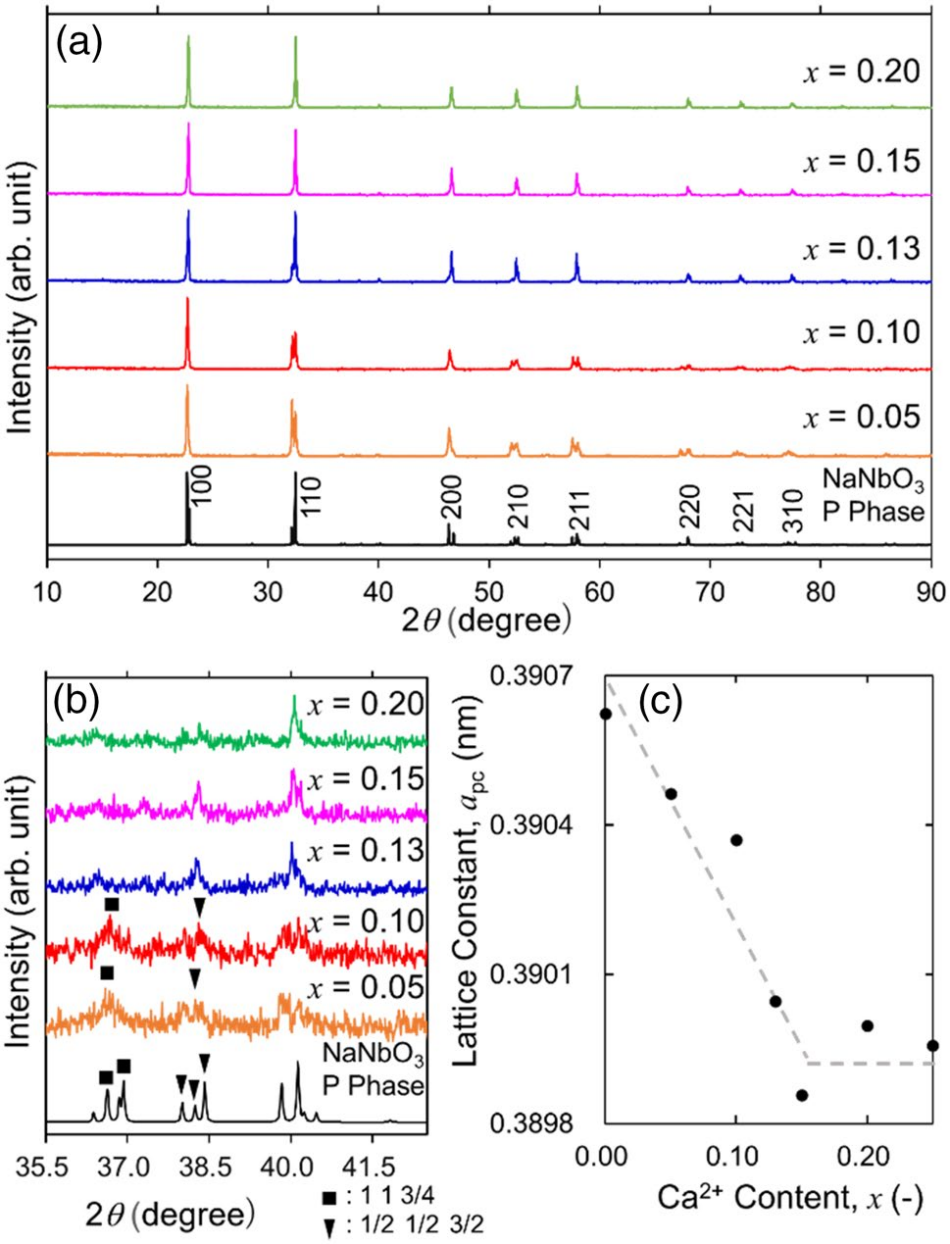
(**a**) XRD patterns of the Ca-modified samples (Ca_*x*_Na_1−2*x*_*V*_*x*_)NbO_3_ and (**b**) enlarged view in the range of 35.5° ≤ 2*θ* ≤ 42.5°. The solid triangles indicate the 1/2 1/2 3/2 reflections and the squares the 1 1 3/4 ones, both of which are characteristic for the AFE-P phase^55^. (**c**) Composition (*x*) dependence of the lattice parameter *a*_pc_ of the pseudocubic unit cell.

Figure 3a shows the XRD patterns of the Ca-modified samples along with that of the AFE-P phase as a reference. Throughout our manuscript, the Miller indices for a 1 × 1 × 1 pseudocubic cell are used. The formation of the perovskite phase was confirmed for all the samples. The intensity of 110 for *x* = 0.05 and 0.10 is relatively small compared with the calculated one of NaNbO_3_, which is likely to be due to a weak preferential orientation of grains in the vicinity of the sample surface. While the splits of the fundamental *hkl* reflections arising from the orthogonal crystal structure were observed for *x* = 0.05 and 0.10, they disappeared for *x* ≥ 0.15. Figure 3b shows an enlarged view of the XRD patterns in the range of 35.5° ≤ 2*θ* ≤ 42.5°. For *x* = 0.05 and 0.10, we found the 1 1 3/4 and 1/2 1/2 3/2 superlattice reflections specific for the AFE-P phase. This result indicates that the AFE-P phase with the antipolar ordering is retained for *x* ≤ 0.10. Though it is difficult to identify the phase for *x* ≥ 0.13, we consider that the AFE-R phase appears (Fig. 1c), which is one of the high-temperature phases of NaNbO_3_ with the space group *Pmmn* or *Pnma* with a 2 × 2 × 6 superlattice structure^15^. This is supported by the temperature dependence of relative permittivity, as discussed later. Figure 3c shows the composition dependence of the lattice parameter of the pseudocubic unit cell (*a*_pc_) obtained from powder XRD patterns (Fig. S2). The ionic radius for 12 coordination is *r*_Na_ = 0.139 nm for Na^+^^54^ and *r*_Ca_ = 0.134 nm for Ca^2+^^54^. In the range of 0 ≤ *x* ≤ 0.15, the *a*_pc_ decreases with increasing *x* and follows the Vegard’s law. This behavior is in contrast to that for Bi-doped NaNbO_3_ with Na vacancy^28,55^ where the cell volume increases with increasing the Bi content^28^. The *a*_pc_ deviates from the Vegard’s law at *x* = 0.15, because a solubility limit of Ca with Na vacancy exists at around *x* = 0.15. It is also notable that our samples have a high insulating property with an extremely low conductivity, e.g., 1.92 $\times$ 10^−11^ S/cm for *x* = 0.10, see Fig. S4. This low conductivity indicates that the charge neutrality in the Ca-modified samples is satisfied by the formation of A-site vacancy rather than a partial reduction of Nb^5+^ to Nb^4+^ inevitably associated with a high conductivity caused by electron injection into the conduction band.

**Figure 4:**
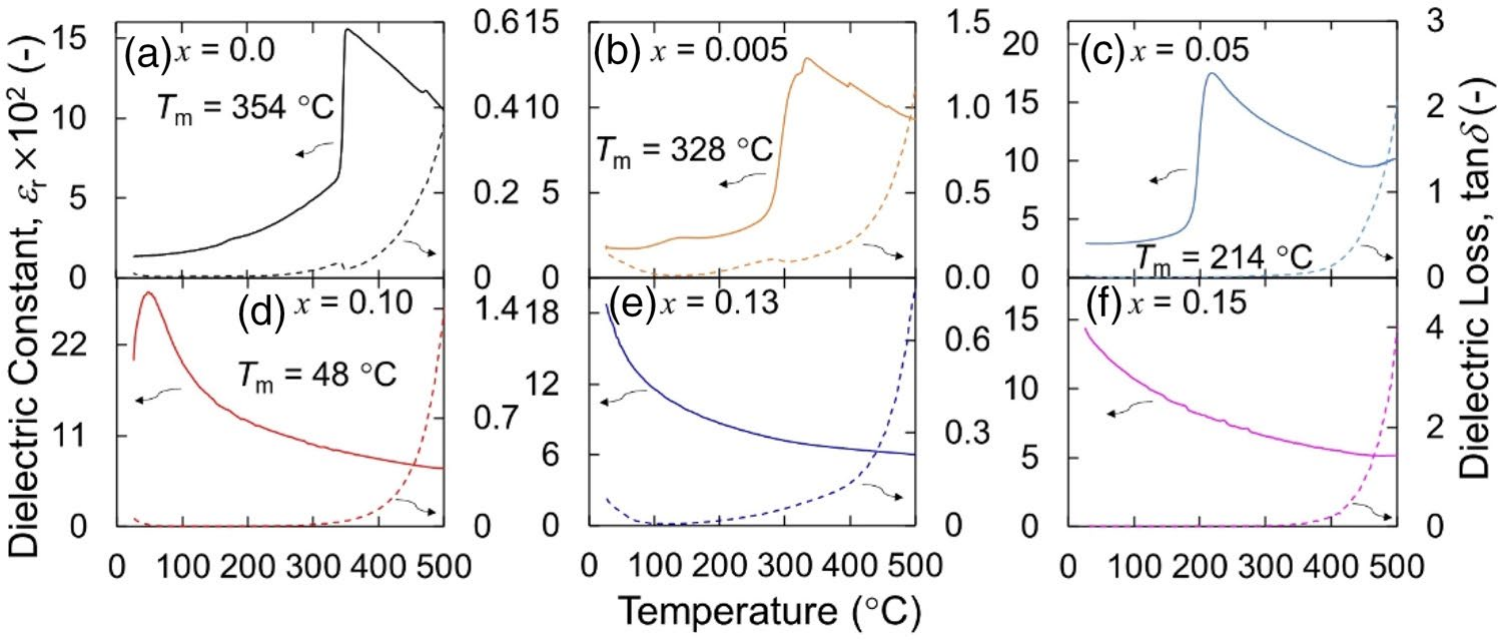
Temperature dependence of the relative dielectric permittivity (*ε*_r_) and loss (tan*δ*) of the Ca-modified samples at 1 kHz: (**a**) *x* = 0, (**b**) *x* = 0.005, (**c**) *x* = 0.05, (**d**) *x* = 0.10, (**e**) *x* = 0.13, and (**f**) *x* = 0.15.

Figure 4 shows the temperature dependence of the relative dielectric permittivity (*ε*_r_) and dielectric loss (tan*δ*). The temperature of the maximum *ε*_r_ (*T*_m_) corresponds to the phase transition from the high-temperature AFE-R phase to the low-temperature AFE-P phase; *T*_m_ = ~ 360 °C for *x* = 0.0^14^. The *T*_m_ decreases with increasing *x* and becomes lower than 25 °C for *x* ≥ 0.13. This *T*_m_ tendency with *x* is consistent with the results of the XRD measurements: the P phase is stabilized for *x* ≤ 0.10 whereas the R phase appears for *x* ≥ 0.13 at room temperature.

**Figure 5:**
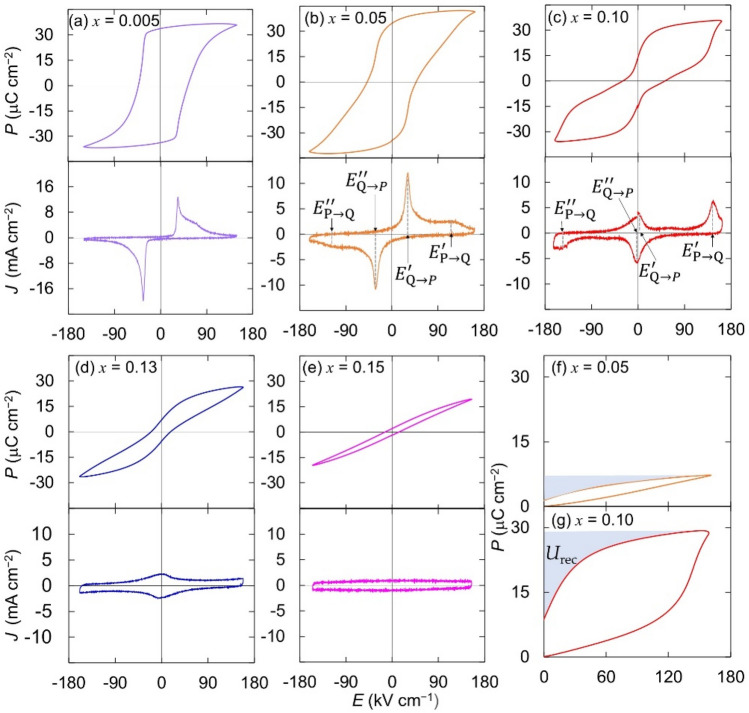
Bipolar *P*-*E* and *J*-*E* hysteresis loops of the Ca-modified samples (Ca_*x*_Na_1−2*x*_*V*_*x*_)NbO_3_: (**a**) *x* = 0.005, (**b**) *x* = 0.05, (**c**) *x* = 0.10, (**d**) *x* = 0.13, (**e**) *x* = 0.15. Unipolar *P*-*E* hysteresis loops at 1 Hz of (**f**) *x* = 0.05 and (**g**) *x* = 0.10.

Figure 5 shows the *P*-*E* and the current density (*J*)-*E* hysteresis properties. These data were obtained for the samples in the poled state, i.e., a unipolar or bipolar *E* was applied prior to the measurements. A typical ferroelectric *P*-*E* loop with a relatively large *P*_r_ of 37 μC cm^−2^ was observed for *x* = 0.005 (Fig. 5a). This is not consistent with the result of the XRD pattern (before the *P*-*E* measurement in Fig. 3) showing the AFE-P phase. The *P*-*E* loop for *x* = 0.05 looks like a ferroelectric polarization hysteresis while that for *x* = 0.10 exhibits an apparent double hysteresis. This seemingly inconsistent with the result of the XRD patterns arises from an irreversible phase transition from the AFE-P to the FE-Q phase for *x* ≤ 0.05, as described later. The sample with *x* = 0.13 exhibits a similar behavior with relaxor antiferroelectrics^56^; a pinched *P*-*E* loop with a small hysteresis and a dielectric dispersion around room temperature (Fig. S3) with a *T*_m_ below 25 °C (Fig. S3). It is reasonable to consider that the AFE-R phase is stabilized for *x* = 0.13. With further increasing *x*, the loop is slanted and eventually almost closed for *x* = 0.15.

**Figure 6:**
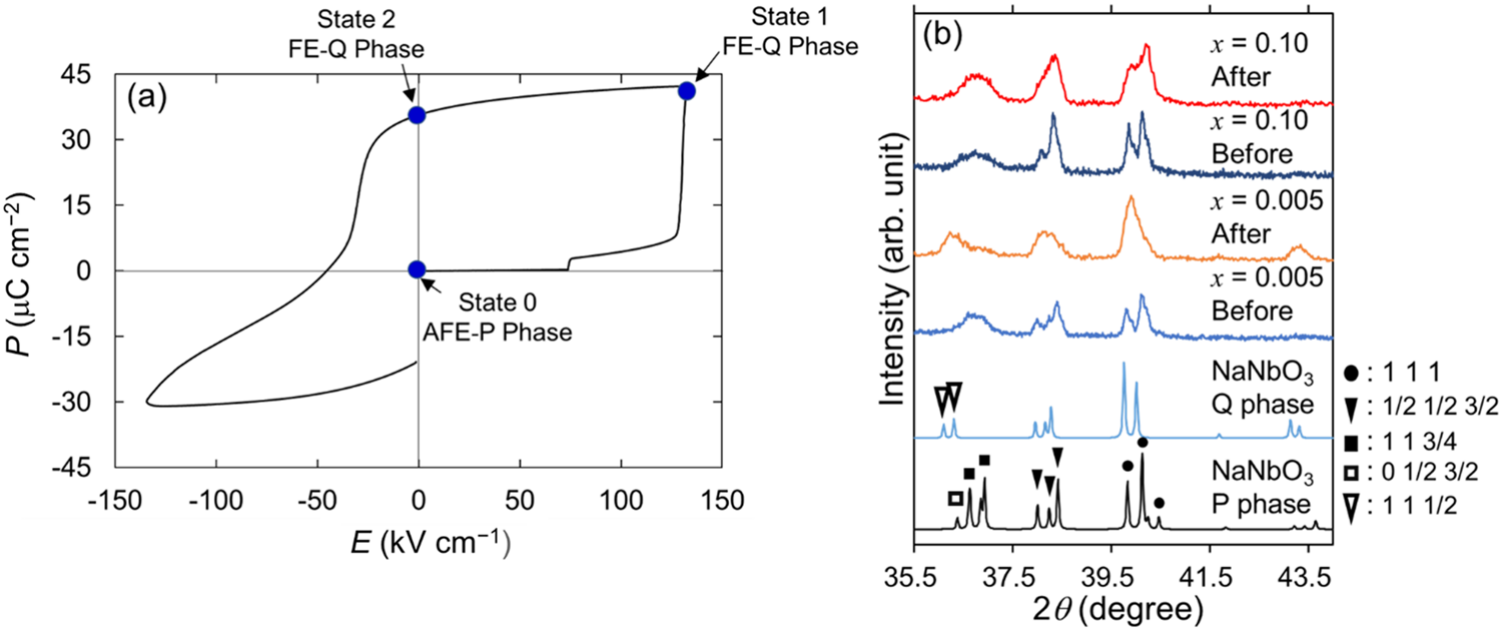
(**a**) First cycle of the bipolar *P*-*E* hysteresis loop for the unpoled sample with *x* = 0.05. (**b**) Micro-XRD patterns of the Ca-modified samples (Ca_*x*_Na_1−2*x*_*V*_*x*_)NbO_3_ with *x* = 0.005, 0.10 after the *P*-*E* measurements. The data for powders with the same compositions are also shown, which are denoted by ‘before’. The solid circles, triangles, and squares indicate 1 1 1, 1/2 1/2 3/2 and 1 1 3/4 reflections^55^, respectively, and the open rectangular and triangles indicate 0 1/2 3/2 and 1 1 1/2 reflection, respectively. The data of NaNbO_3_ in the P and Q phases are referenced from 00–033–1270^51^ and 01–079–7429^52^ in ICDD, respectively.

Figure 6a shows the first cycle of the bipolar *P*-*E* hysteresis loop for an as-prepared, unpoled sample (*x* = 0.05). With a positive sweep of *E*, the sample exhibits a jump of *P* at a *E*_T_ and has a large *P* in State 1. This large *P* is retained even after removing *E* (State 2). With a negative *E* sweep followed by turning off the field, the sample displays an apparent negative *P*. As described later, the overall behavior of the first *P*-*E* loop can be explained by the irreversible phase transition from the AFE-P phase (State 0) to the FE-Q phase (States 1 and 2).

Figure 6b shows the XRD patterns after the *P*-*E* measurements (denoted by ‘after’) along with those for the powders with the same compositions (‘before’). For *x* = 0.005 (before), the 1 1 3/4 superlattice reflection specific for the AFE-P phase^57^ was observed. Moreover, the 1 1 1 reflection splits in the same manner as the reference of the P phase. After the measurements, the 1 1 3/4 reflection disappears while the 1 1 1/2 reflection of the FE-Q phase appears at 2*θ* = 36.2°^52^. These results show that once an *E* exceeding its *E*_T_ is applied a phase transition from the P to the Q phase takes place and also that the Q phase remains stable even after removing *E*. The well-opened *P*-*E* loop for *x* = 0.005 (Fig. 5a) arises from the polarization switching of the FE-Q phase.

In contrast, the XRD data for *x* = 0.10 (after) represents the P-phase feature with the 1 1 3/4 and 1/2 1/2 3/2 reflections. These results along with the double hysteresis loop (Fig. 5c) clearly demonstrate that the sample (*x* = 0.10) displays a reversible *E*-induced P-Q phase transition. The *J*-*E* loop has two broad peaks in the higher *E* region as well as two sharp peaks in the lower *E* region. We define the *E* values for the former two peaks corresponding to the transition from the P to the Q phase as ${E}_{\mathrm{P}\to \mathrm{Q}}^{\prime}$ and ${E}_{\mathrm{P}\to \mathrm{Q}}^{\prime \prime}$ and those for the latter peaks corresponding to the transition from the Q to the P phase as ${E}_{\mathrm{Q}\to \mathrm{P}}^{\prime}$ and ${E}_{\mathrm{Q}\to \mathrm{P}}^{\prime \prime}$. The threshold fields of the *E*-indued phase transition, ${E}_{\mathrm{P}\to \mathrm{Q}}$ and ${E}_{\mathrm{Q}\to \mathrm{P}}$, for *x* = 0.05 and 0.10 are summarized in Table 1.

**Table 1:** Threshold fields of the *E*-induced phase transition between the AFE-P and the FE-Q phases estimated from the *J*–*E* loops (Fig. 5).

*x*	${E}_{\mathrm{P}\to \mathrm{Q}}=(\left|{E}_{\mathrm{P}\to \mathrm{Q}}^{\prime}\right|+\left|{E}_{\mathrm{P}\to \mathrm{Q}}^{\prime \prime}\right|)/2$ (kV cm^−1^)	${E}_{\mathrm{Q}\to \mathrm{P}}=(\left|{E}_{\mathrm{Q}\to \mathrm{P}}^{\prime}\right|+\left|{E}_{\mathrm{Q}\to \mathrm{P}}^{\prime \prime}\right|)/2$ (kV cm^−1^)
0.05	110	31.5
0.10	148	1.5

With increasing *x*, ${E}_{\mathrm{P}\to \mathrm{Q}}$ rises while ${E}_{\mathrm{Q}\to \mathrm{P}}$ approaches zero. It is considered that the double *P*-*E* loop for *x* = 0.10 is a result of the small ${E}_{\mathrm{Q}\to \mathrm{P}}$. It is expected that a higher Ca-*V* content, i.e., a further increase in *x*, gives rise to a clearer double loop. However, for the samples with *x* ≥ 0.13, the polarization switching derived from the *E*-induced phase transition was not observed. This is because the AFE-R phase is stabilized and then the ${E}_{\mathrm{P}\to \mathrm{Q}}$ becomes higher than its breakdown field. It is also noted that the peaks of *J* for the P → Q transition at ${E}_{\mathrm{P}\to \mathrm{Q}}^{\prime}$ and ${E}_{\mathrm{P}\to \mathrm{Q}}^{\prime \prime}$ are sharp compared with those for the Q → P transition at ${E}_{\mathrm{Q}\to \mathrm{P}}^{\prime}$ and ${E}_{\mathrm{Q}\to \mathrm{P}}^{\prime \prime}$, especially for *x* ≤ 0.05. These phase transitions take place through nucleation and growth dynamics of FE (Q) domains in the AFE (P) matrix and AFE (P) domains in the FE (Q) one. We think that local random fields, as reported in NaNbO_3_–(Bi_0.5_Na_0.5_)TiO_3_ solid solutions^58^, play an important role in the *E*-induced phase transitions, but the details are unclear.

Figure 5f,g show the unipolar *P*-*E* hysteresis loops. The recoverable energy storage density (*U*_rec_) and the energy efficiency (*η*) are calculated by the following equations.

1 $${U}_{\mathrm{rec}}={\int }_{{P}_{\mathrm{r}}}^{{P}_{\mathrm{max}}}EdP,$$

2 $$\eta =\frac{{U}_{\mathrm{rec}}}{{U}_{\mathrm{rec}}+{U}_{\mathrm{loss}}}\times 100,$$

where *P*_max_, *P*_r_, and *U*_loss_ are maximum polarization, remanent polarization, and energy loss, respectively^1,4^. The *U*_rec_ and the *η* estimated from the hysteresis loops are 0.34 J cm^−3^ and 34% for *x* = 0.05 and 0.74 J cm^−3^ and 17% for *x* = 0.10. The larger *U*_rec_ for *x* = 0.10 is attributed to the reversible *E*-induced phase transition between the AFE-P and the FE-Q phases.

**Figure 7:**
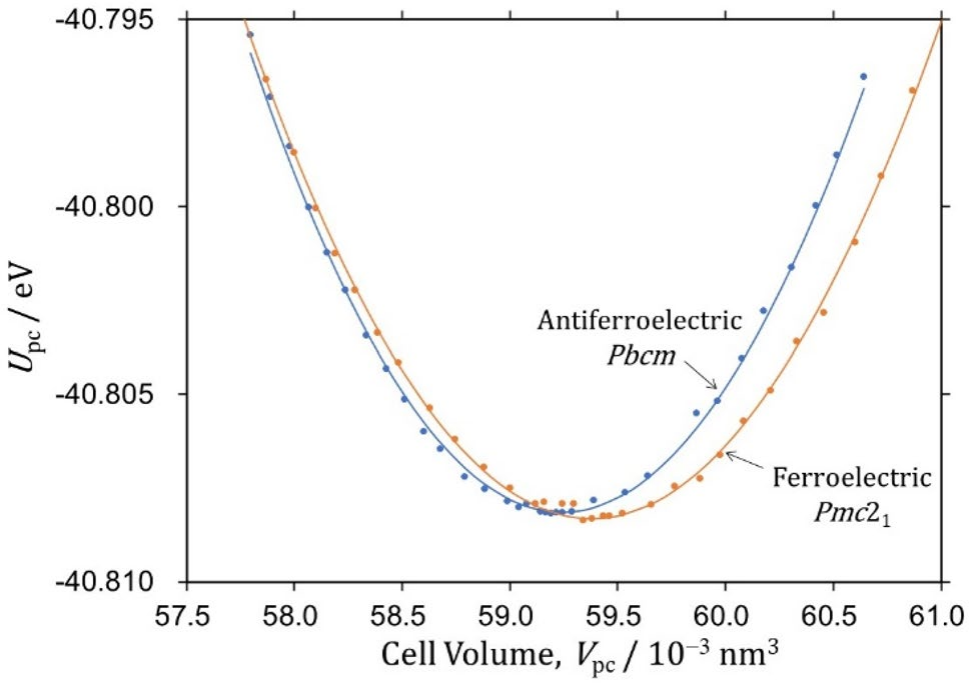
Pseudocubic total energy (*U*_pc_) per the ABO_3_ formula unit as a function of pseudocubic unit cell volume (*V*_pc_) for the AFE-P phase (*Pbcm*) and the FE-Q phase (*Pmc*2_1_). The hydrostatic pressure (*p*) changes between −2.8 GPa and 2.8 GPa. Filled circles indicate the data obtained by DFT calculations and solid lines the fitting curves obtained by a nonlinear least square method using Eq. (3).

Figure 7 exhibits the total energy *U*_pc_ of NaNbO_3_ as a function of pseudocubic unit cell volume *V*_pc_ in the range of hydrostatic pressure *p* between −2.8 GPa and 2.8 GPa. It is interesting to note that the difference in *U*_pc_ is relatively small; several meV at a smaller *V*_pc_ and ~ 10 meV at a larger *V*_pc_. When the positive *p* is applied, i.e., the *V*_pc_ is compressed, the *U*_pc_ of the AFE-P phase is slightly smaller. In contrast, at a negative *p* with an expanded *V*_pc_, the FE-Q phase is markedly stabilized.

**Figure 8:**
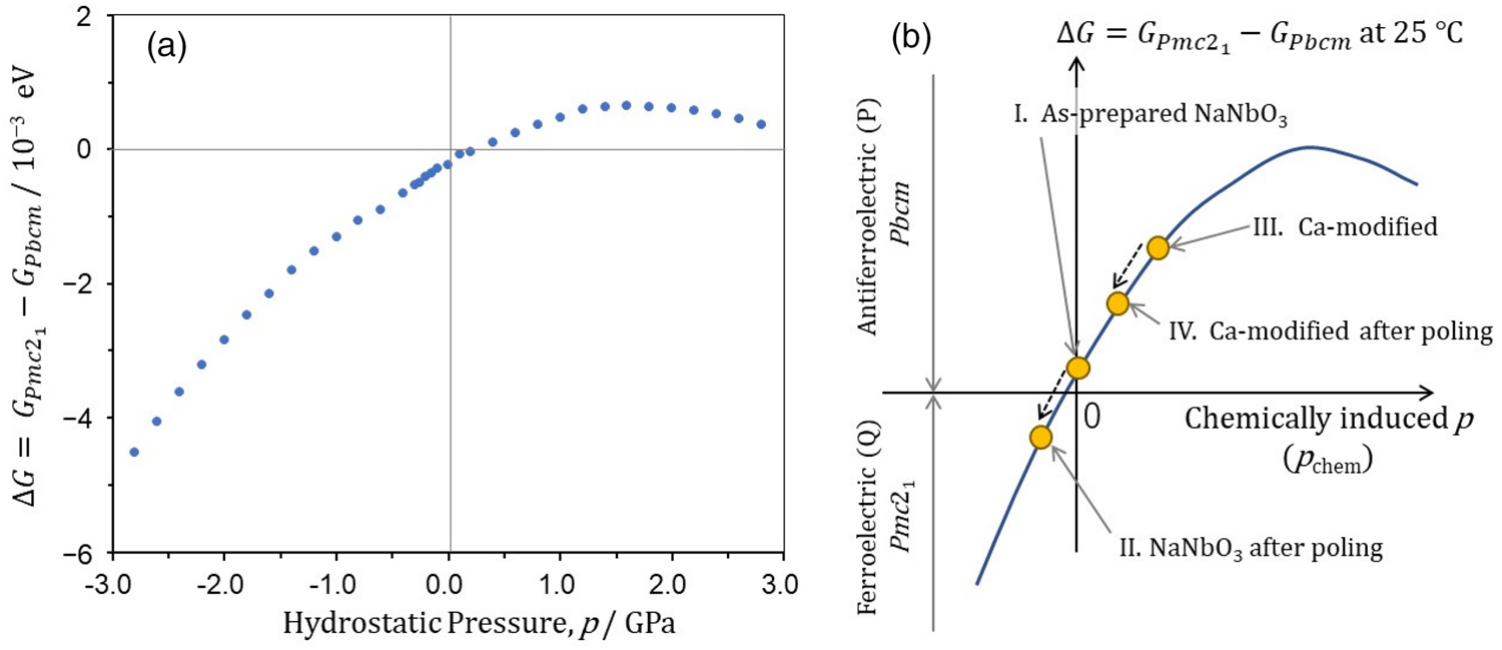
Free energy difference ($\Delta G {= G}_{Pmc{2}_{1}}-{G}_{Pbcm}$), where ${G}_{Pmc{2}_{1}}$ and ${G}_{Pbcm}$ denotes the free energies of the FE-Q phase (*Pmc*2_1_) and the AFE-P phase (*Pbcm*) one: (**a**) obtained by DFT calculations for NaNbO_3_ at zero kelvin and (**b**) aligned for the ceramic samples at 25 ℃. The AFE-P phase is stabilized in the region of ∆*G* > 0 while the FE-Q phase appears in the region of ∆*G* < 0. In reality, the Ca ions and the vacancies on the A site play a different role, while the FE and AFE orderings stem primarily from an orbital interaction between Nb-4*d* and O-2*p*. We assume that the effects of the Ca modification on $\Delta G$ for our samples is simplified by the change in chemically induced *p* (*p*_chem_) in (**b**).

Figure 8a displays the free-energy difference of ${\Delta G = {G}_{{Pmc2}_{1}}-G}_{Pbcm}$ vs. *p* ($G = {U}_{\mathrm{pc}}+p{V}_{\mathrm{pc}}$). The ∆*G* curve is not smooth caused by the Pulay stress^59–61^; the plane-wave basis set is not complete with respect to changes in the volume. In the *p* range, *p* > 0.2 GPa, ∆*G* is positive and the AFE-P phase appears. In the range of *p* < 0.2 GPa, ∆*G* becomes negative and the FE-Q phase arises.

## Discussion

It is important to note that the ∆*G* feature in Fig. 8a is the result of the DFT calculations at zero kelvin under the periodic boundary condition. In reality, our experiments were performed at 25 °C. Additionally, our samples underwent the phase transitions during cooling from the high-temperature cubic phase to the low-temperature orthorhombic phases. Moreover, the samples were cooled down to room temperature under the constraints of a spatially fixed matrix of dense ceramics. We therefore think that the zero point of the chemically induced *p* (*p*_chem_) of the as-prepared NaNbO_3_ sample at 25 ℃ (Fig. 8b) is inevitably shifted from that in Fig. 8a.

Figure 8b schematizes the ∆*G* curve with respect to *p*_chem_ for our samples, where the effects of the Ca modification is treated as the change in *p*_chem_. The as-prepared undoped sample is of the AFE-P phase and placed at zero *p*_chem_ (I.). The electrical poling, an application of *E*, induces an irreducible phase transition to the FE-Q phase, which is accompanied by the marked change in lattice parameters. It is reasonable to consider that this electrical poling causes a shift of *p*_chem_ to the region where the FE-Q phase arises (II.), probably because of the influence of intragranular stress^53^.

Here, we discuss the effect of the Ca modification on *p*_chem_. As shown in Fig. 3c, the increase in *x* (the Ca-*V* content) decreases *a*_pc_. In other words, the Ca modification increases *p*_chem_ as a result of a reduced *V*_pc_, and the sample with *x* = 0.10 moves at III., where the AFE-P phase appears. It is likely that the electrical poling decreases *p*_chem_; the poled sample is still of the AFE-P phase (IV.). We show that the positive *p*_chem_ by the Ca modification stabilizes the AFE-P phase even after the electrical poling and is thereby effective for realizing the reversible *E*-induced phase transition, as observed for the sample (*x* = 0.10).

In conclusion, we have investigated *E*-induced phase transitions in Ca-modified NaNbO_3_ ceramics with A-site vacancy [(Ca_*x*_Na_1−2*x*_*V*_*x*_)NbO_3_]. We experimentally found that the Ca modification reduces a cell volume and the resultant positive *p*_chem_ stabilizes the AFE-P phase for *x* ≤ 0.10. The reversible *E*-induced phase transition between the AFE-P and the Q phases occurs for the sample with *x* = 0.10, which results in a double hysteresis loop. The *U*_rec_ of the sample (*x* = 0.10) with the reversible phase transition is more than two times as high as that of the sample (*x* = 0.05) with the irreversible one. Our study opens the way to utilizing *p*_chem_ for NaNbO_3_-based antiferroelectric materials for energy storage applications.

## Methods

### Material preparation

Undoped NaNbO_3_ and Ca-modified NaNbO_3_ [(Ca_*x*_Na_1−2*x*_*V*_*x*_)NbO_3_] ceramic samples with *x* = 0.005, 0.05, 0.10, 0.13, 0.15, and 0.20 were prepared by a solid-state reaction using Na_2_CO_3_ (99.99%), CaCO_3_ (99.99%), and Nb_2_O_5_ (99.99%) as raw materials. Na_2_CO_3_ powders were dried at 280 °C for at least 4 h before weighing. The raw materials were mixed by ball milling for 1 h in ethanol. The mixed powders were dried and calcined in an alumina crucible at 900 °C for 3 h in air. The resultant powders were pulverized by ball milling for 1 h and pressed into pellets with a diameter of 10 mm followed by sintering at 1250–1300 °C for 4–5 h in air for *x* = 0 and in an oxygen atmosphere for the other compositions. To suppress the volatilization of Na, the pellets were covered by powders with the same composition. The relative densities of the samples are as follows: 92.4% for NaNbO_3_ and 94.9% for *x* = 0.005, 98.2% for *x* = 0.05, 98.3% for *x* = 0.10, 98.9% for *x* = 0.13, 98.7% for *x* = 0.15, and 97.3% for *x* = 0.20. The samples obtained were cut and polished into disks with a thickness of 0.15–0.20 mm. An annealing for oxidation was performed at 1000 °C for 48 h in air. For the measurements of relative permittivity and *P*-*E* hysteresis loops, Au electrodes with a diameter of 1 mm and 2 mm, respectively, were sputtered on both sides of the disks. The lattice parameters were obtained by the Rietveld analysis for powder XRD patterns (Fig. S2) with a Rietveld refinement program Z-Rietveld (version 1.1.3). We adopted space group *Pbcm* (the AFE-P phase) and *Pnma* (the AFE-R phase). Structural refinement was conducted until the reliability index *R*_wp_ becomes minimum. The *a*_pc_ is calculated by ${a}_{\mathrm{pc}}= \sqrt[3]{(a\times b\times c)/8}$ for *Pbcm* and ${a}_{\mathrm{pc}}= \sqrt[3]{(a\times b\times c)/24}$ for *Pnma*, respectively, where *a*, *b*, and *c* are the refined lattice parameters of the orthorhombic unit cell. For the powder samples for the Rietveld analysis, the calcined powders were pulverized by ball milling for 1 h and then sintered at 1150–1300 °C for 4 h in air.

### DFT calculations

DFT calculations were performed via the generalized gradient approximation^62^ with a plane wave basis set. The projector-augmented wave method^63^ was applied by the Vienna ab initio simulation package (VASP)^64^. We employed the gradient-corrected exchange-correlation functional of the Perdew-Burke-Ernzerhof revised for solids (PBEsol)^65^ and a plane-wave cut-off energy of 520 eV. The following two phases with different symmetries were considered: the AFE-P phase with space group *Pbcm* (Z = 8) and the FE-Q phase with space group *Pmc*2_1_ (Z = 4). The adopted mesh size of the k-point sampling grid was 9 × 9 × 7 for *Pbcm* and 11 × 11 × 11 for *Pmc*2_1_. We confirmed that the optimized structures and the resultant total energies were unchanged when the finer *k*-point mesh sizes were adopted.

To evaluate the phase stability, we calculated the total energy (*U*) as a function of the cell volume (*V*) and then analyzed by the Murnaghan equation of state^66^

3 $$U\left(V\right)={U}_{0}+\frac{{B}_{0}V}{{B}_{0}^{\mathrm{^{\prime}}}}\left[\frac{{\left({V}_{0}/V\right)}^{{B}_{0}^{\mathrm{^{\prime}}}}}{{B}_{0}^{\mathrm{^{\prime}}}-1}+1\right]-\frac{{B}_{0}{V}_{0}}{{B}_{0}^{\mathrm{^{\prime}}}-1} ,$$

where *U*_0_, *B*_0_, *B*_0_′, and *V*_0_ are the total energy, the bulk modulus and its first derivative with respect to the hydrostatic pressure (*p*) and V at *p* = 0. We converted the *U* and the *V* into the *U*_pc_ and the *V*_pc_ of the pseudocubic unit cell, because the cell sizes of the two phases are different. Since the free energy (*G*) is expressed as *G* = *U*_pc_ + *pV*_pc_, we can obtain the relation between *G* and *p* using the fitting parameters in Eq. (3). It should be noted that our DFT calculations predict the *G* feature at zero kelvin.

## Supplementary Information

Supplementary Figures.

## Data Availability

Any data generated from these studies is available from the corresponding authors upon reasonable requests.
