# Supplementary material for: Reversible electric-field-induced phase transition in Ca-modified NaNbO3 perovskites for energy storage applications
Source: Sci Rep. 2023 Apr 25;13:6771. doi: 10.1038/s41598-023-33975-6 (PMC10130038; doi:10.1038/s41598-023-33975-6)
Supplement: Supplementary file 1 — Supplementary Figures. [file 41598_2023_33975_MOESM1_ESM.docx]

**Supplementary Information**

**Reversible electric-field-induced phase transition in Ca-modified NaNbO_3_ perovskites for energy storage applications**

# Seiyu Aso1, Hiroki Matsuo2,* and Yuji Noguchi3,*

1 Department of Computer Science and Electrical Engineering, Graduate school of Science and Technology, Kumamoto University, 2-39-1, Kurokami, Chuo-ku, Kumamoto 860-8555, Japan

2 International Research Organization for Advanced Science & Technology (IROAST), Kumamoto University, 2-39-1, Kurokami, Chuo-ku, Kumamoto 860-8555, Japan

3 Division of Information and Energy, Faculty of Advanced Science and Technology, Kumamoto University, 2-39-1, Kurokami, Chuo-ku, Kumamoto 860-8555, Japan

*Correspondence: matsuo_h@cs.kumamoto-u.ac.jp and yuji19700126@gmail.com

| 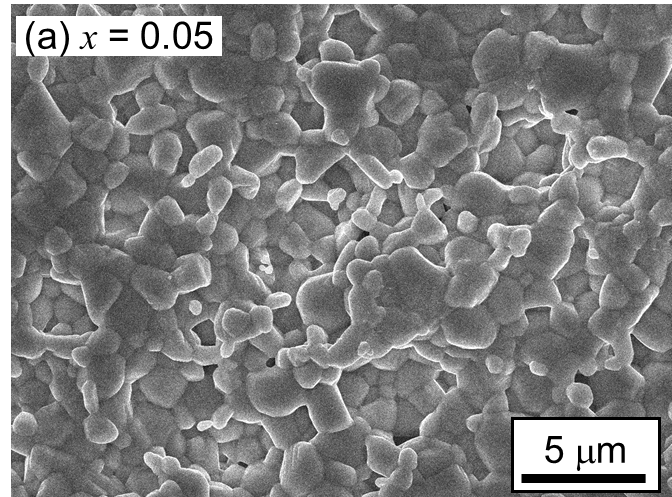 | 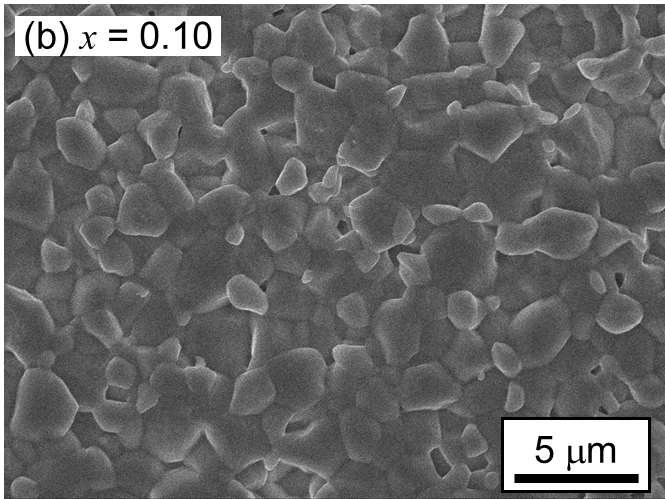 |
| --- | --- |
| 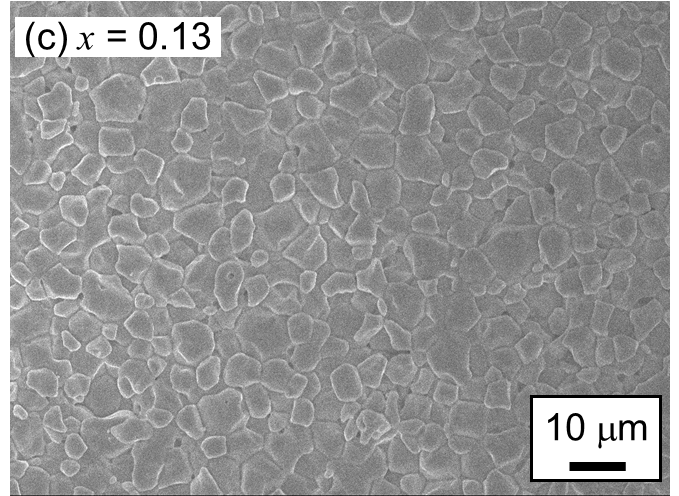 | 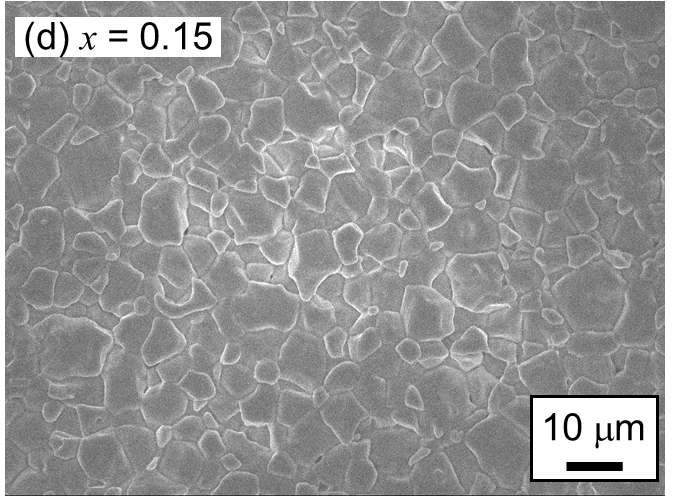 |
| 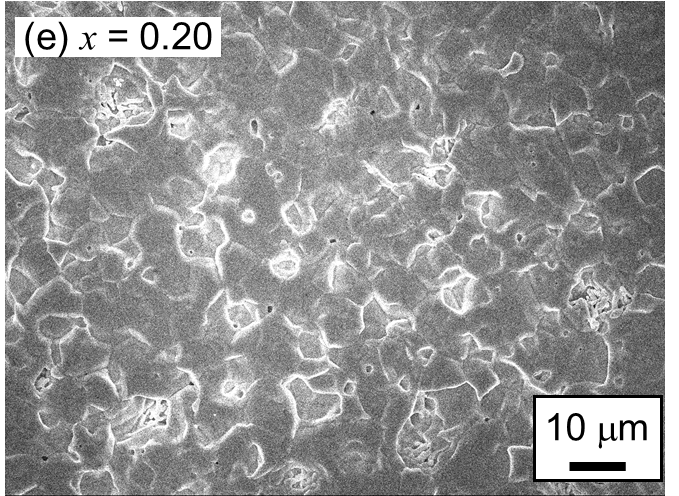 | |

**Figure S1.** Cross sectional SEM images of Ca-NaNbO_3_ ceramics with *x* of (a) 0.05, (b) 0.10, (c) 0.13, (d) 0.15, and (e) 0.20. Composition dependence of average grain size of the samples in Fig. 1(f) was obtained using these SEM images.


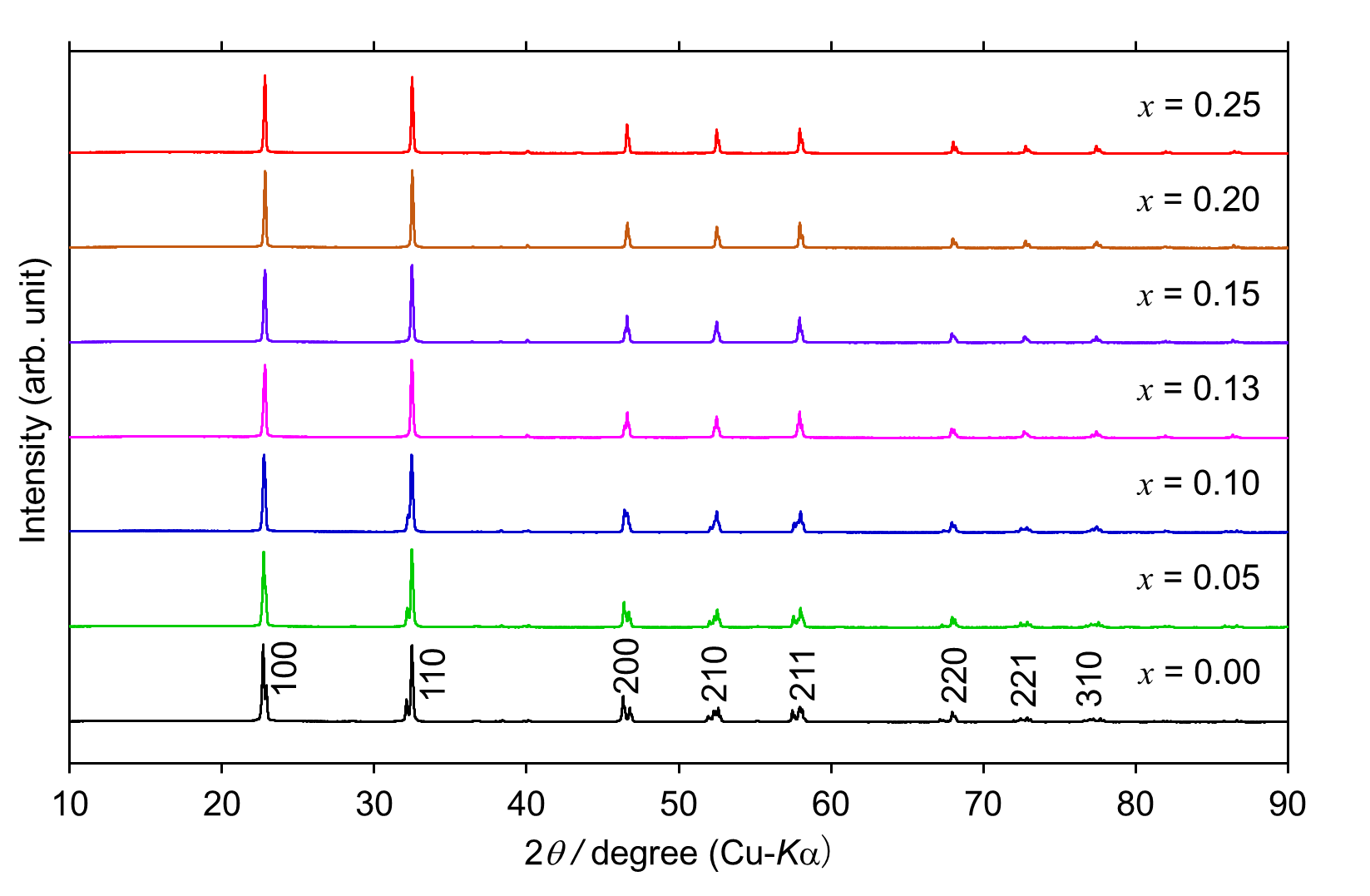


**Figure S2.** XRD patterns of the Ca-modified NaNbO_3_ powders. Composition dependence of lattice parapeters of the samples in Fig. 3(c) was obtained by the Rietveld analysis for these XRD patterns. The lattice parameters with *x* = 0.13 and 0.25 have been also added in Fig. 3(c) in original manuscript.


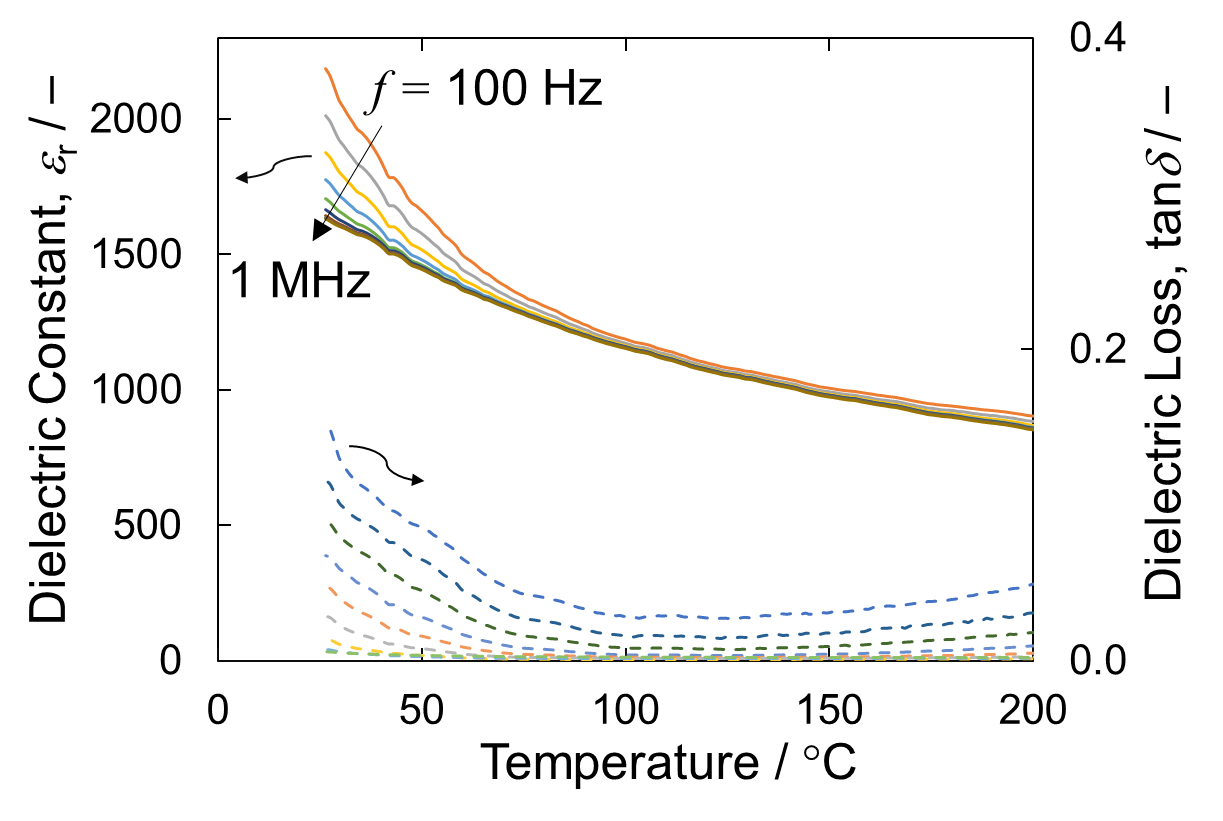


**Figure S3**. Temperature dependence of relative dielectric permittivity and dielectric loss (tan *δ*) of the Ca-modified sample for *x* = 0.13 measured at 100 Hz, 300 Hz, 1 kHz, 3 kHz, 10 kHz, 30 kHz, 100 kHz, 300 kHz, and 1 MHz.

_
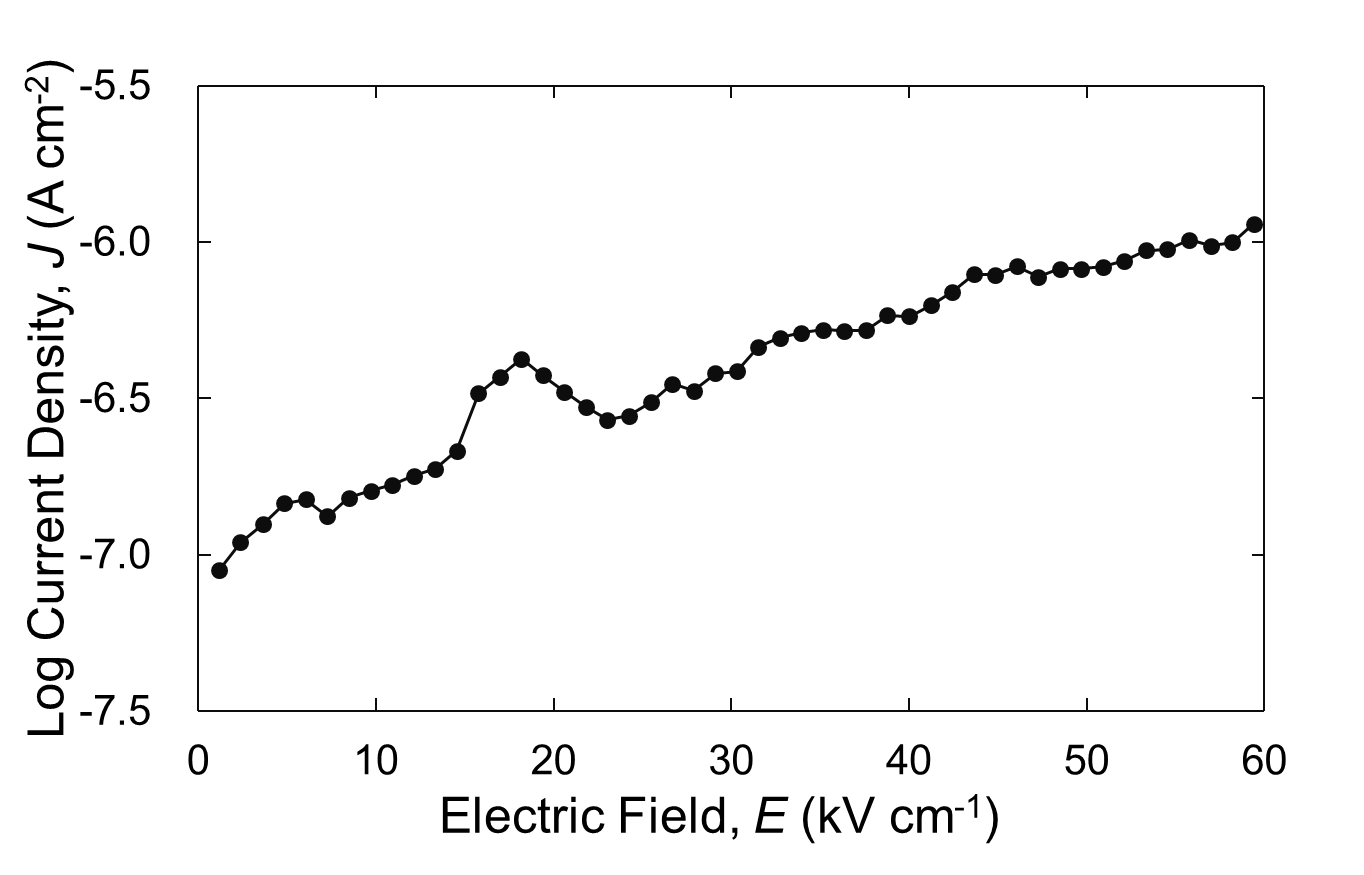
_

**Figure S4.** Current density of the Ca-modified sample with *x* = 0.10.
